# Strong‐Gradient Diffusion‐Weighted Imaging of Prostate Cancer Using an Inside‐Out Nonlinear Gradient Coil

**DOI:** 10.1002/mrm.70252

**Published:** 2026-01-08

**Authors:** Horace Z. Zhang, Nahla M. H. Elsaid, Terence W. Nixon, Andrew Dewdney, Dana C. Peters, Jeffrey C. Weinreb, Preston C. Sprenkle, R. Todd Constable, Gigi Galiana

**Affiliations:** ^1^ Department of Biomedical Engineering Yale University New Haven Connecticut USA; ^2^ Department of Radiology & Biomedical Imaging Yale University New Haven Connecticut USA; ^3^ Division of Diagnostic Imaging & Radiology Children's National Health System Washington DC USA; ^4^ Siemens Healthineers AG Erlangen Germany; ^5^ Department of Urology Yale University New Haven Connecticut USA

**Keywords:** diffusion MRI, gradient, prostate

## Abstract

**Purpose:**

To demonstrate improved image quality and lesion conspicuity in prostate diffusion‐weighted imaging (DWI) using an inside‐out nonlinear gradient coil that provides locally strong gradients (200–500 500 mT/m) at typical prostate positions.

**Theory and Methods:**

Before applying the nonlinear gradient coil to DWI with Echo Planar Imaging (EPI) readout, we investigated geometric distortion and eddy currents, and proposed necessary corrections. We then developed two DWI protocols (*b*_max_ = 1000 and 3000 s/mm^2^) with minimized echo time (TE) and tested them on volunteers and patients. We validated apparent diffusion coefficient (ADC) maps from the nonlinear gradient acquisition against the reference (linear gradients only). We quantified improvements in signal‐to‐noise ratio (SNR), lesion contrast‐to‐noise ratio (CNR), and lesion‐to‐normal‐tissue contrast ratio in the compartmental map of restricted diffusion.

**Results:**

Corrections effectively reduced nonlinear‐gradient DWI artifacts. ADC maps from linear‐ and nonlinear‐gradient‐encoded studies agreed well, with a normalized root‐mean‐square‐error of ∼10%, a common level of ADC variation. TE was significantly reduced from 57 to 42–47 ms for moderate *b*‐values (≤ 1000 s/mm^2^) and from 72 to 42–54 ms for high *b*‐values (≤ 3000 s/mm^2^). Consequently, SNR increased by 3%–38% (median 16%, *p* < 0.01) and 7%–38% (median 26%, *p* < 0.01), respectively. Lesion CNR improved by a median of 133% at *b* = 2000 s/mm^2^ and 217% at *b* = 3000 s/mm^2^. The restricted diffusion component in lesions was more conspicuous at short TE, with a median 23% increase in lesion‐to‐normal‐tissue contrast ratio (*p* = 0.02).

**Conclusion:**

The inside‐out nonlinear gradient coil enhances prostate DWI.

## 1 Introduction

There have been numerous efforts pursuing higher gradient strengths for diffusion‐weighted imaging (DWI) [1, 2]. One direct benefit of higher gradient strengths is faster diffusion encoding, and thus reduced echo times (TE), which increases signal‐to‐noise ratio (SNR) by preserving signals from exponential decay and captures signal from clinically important short‐*T*_2_ compartments. Furthermore, shorter diffusion‐sensitizing gradient pulses probe finer microstructure [3]. High‐performance whole‐body gradients have shown promising results in the brain [4, 5], prostate [6, 7, 8, 9], cardiac [10, 11] imaging. However, the increased inductance from additional windings and greater risk of peripheral nerve stimulation pose challenges [2]. Above all, these gradient systems are expensive and primarily limited to research, e.g., Connectome 1.0 (300 mT/m) [2]. The only commercially available whole‐body product in this class is Cima.X (200 mT/m) [12], which secured FDA clearance in early 2024.

Organ‐specific gradients, most notably head gradients, can achieve much stronger gradient strength due to the smaller radius [13, 14, 15, 16, 17, 18, 19]. Single‐sided gradients have also been considered as a strategy to generate strong diffusion weighting over a targeted field of view. Examples include a single‐sided planar coil for spine DWI with nonlinearity correction [20], a butterfly coil under the neck optimized for carotid DWI [21], a curved single‐sided magnet for fat quantification of liver [22], and a breast gradient coil generating gradient up to 1 T/m [23].

**FIGURE 1:**
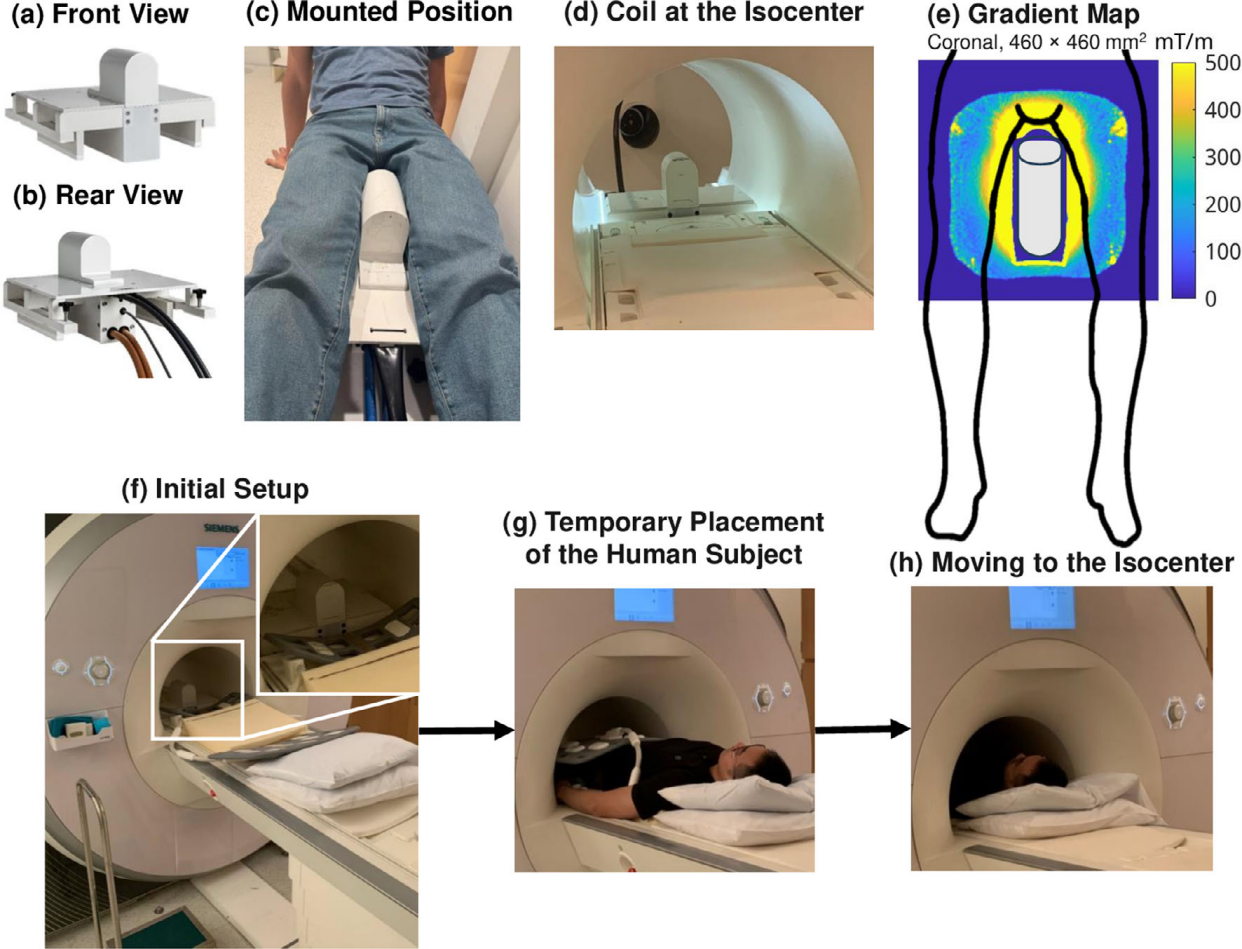
(a, b) The front and rear views of the inside‐out nonlinear gradient coil. (c) Mounted position of legs straddling the coil. (d) The coil is positioned at the isocenter of the scanner bore. (e) The gradient map in a coronal plane. (f–h) Pipeline for positioning the human subject. (f) The initial setup of the nonlinear gradient coil, the receiving coils, and the cushion. (g) The subject's pelvis is brought close to the nonlinear gradient coil while maintaining the posture shown in (c). (h) The subject is moved to the isocenter simultaneously with the nonlinear gradient coil, which is then fixed to the rail by tightening the bolt.

For prostate DWI, we proposed an inside‐out nonlinear gradient which sits in the center of the magnet bore between the patient's legs [24], shown in Figure 1a–c. The lightweight (15 kg) compact prototype is a cylinder with a diameter of 10 cm and a length of 15 cm. It can be installed by one person in under a minute, making it suitable for on‐demand installation between scan sessions. Reproducible positioning is achieved by threaded holes on the sliding rail of the scanner bore that mate with bolts on the gradient housing [25] (Figure 1d).

Compared to high‐power linear whole‐body gradients, the lower inductance reduces the challenges associated with slew rate or voltage, and the limited spatial coverage diminishes the potential for peripheral nerve stimulation. The geometry is suitable for prostate imaging at the front face of the cylinder and leg imaging on the side. The coil is driven by a 600 A amplifier, derated to a peak voltage of 500 V. The locally strong gradient is more than 800 mT/m immediately in front of the coil and reaches 200–500 mT/m at typical prostate locations (Figure 1e). Because the nonlinear gradient operates in tandem with the linear gradients, it increases the available gradient strength at any drive current.

The clinical motivation for the inside‐out nonlinear gradient coil is its potential to improve prostate DWI. Prostate cancer is the second leading cause of cancer‐related death in men [26], and the combination of high prevalence and sometimes slow progression creates a critical need for accurate imaging [27, 28]. Multiparametric MRI [29, 30] is now recognized as a crucial step of prostate cancer diagnosis, both for early detection and for monitoring to avoid overtreatment. Within the multiparametric protocol, DWI and ADC are considered dominant contrasts for diagnosis because they reflect the mobility of water molecules within tissue. In prostate cancer, the increased cellular density and reduced luminal space restrict water diffusion, leading to high signal intensity on DWI and low ADC values. These features make DWI/ADC particularly sensitive to malignant changes in prostate tissue [31, 32]. Specifically, the ADC map is typically derived from a DWI series with moderate diffusion weighting (*b*‐value ≤ 1000 s/mm^2^). In addition, DWI with strong diffusion weighting (*b*‐value > 1000 s/mm^2^) can separate equivocal compartmental signals [33] and increase lesion visibility [7, 34].

An emerging field for prostate cancer diagnosis is multicompartmental diffusion modeling, which usually decomposes the DWI signal into a linear combination of exponential decays. Prominent methods include RSI(restriction spectrum imaging) [35, 36] and VERDICT(vascular, extracellular and restricted diffusion for cytometry in tumors) [37, 38]. Other models consider time‐dependent diffusivity, such as IMPULSED (Imaging Microstructural Parameters Using Limited Spectrally Edited Diffusion) [39], which has been applied to microstructural mapping of prostate cancer [40]. In each of these models, an increased contribution from short‐*T*_2_ low‐ADC compartments is a hallmark of prostate cancer, reflecting a greater signal fraction originating from epithelial tissue. This framework has also been validated in results from hybrid multidimensional MRI [41, 42, 43]. Sensitivity to these short‐*T*_2_ lesions is enhanced by reduced TE achievable with strong gradient systems, such as the inside‐out nonlinear gradient coil.

In this study, we improved prostate DWI with the inside‐out nonlinear gradient coil for diffusion encoding. We first characterized the coil performance for DWI with echo‐planar imaging (EPI), including assessments of susceptibility artifacts and eddy currents, and proposed artifact correction methods. The improved DWI protocol was then applied in healthy subjects as well as patients with suspected prostate cancer to evaluate its impact on image quality and lesion conspicuity. We hypothesized that the strong, localized gradients would enable shorter TE, thereby enhancing SNR and improving conspicuity of prostate lesions with short‐*T*_2_ low‐ADC compartments.

## 2 Theory

### 2.1 Background Field With the Nonlinear Gradient Coil

The nonlinear gradient coil, being a large mass of copper at the center of the scanner bore, causes significant inhomogeneity in the static background field. This has two direct consequences for EPI, namely distortion and ghosting. Distortion is usually mitigated by parallel imaging [44, 45], multi‐shot EPI [46, 47, 48], reduced field of view [49, 50, 51], or slice‐by‐slice shimming [52, 53, 54]. It can also be corrected in processing by acquiring opposite phase‐encoding directions [55, 56]. Ghosting artifacts are usually addressed by one‐dimensional linear phase correction (1D LPC) [57], as well as advanced methods that factor in phase variations between odd/even lines [58], shots [48], or both [59]. The *N*/2 ghosts are further influenced by imperfect parallel imaging reconstruction and eventually appear as *N*/(2 × *R*) ghosts, where *R* is the in‐plane acceleration factor [60].

The inhomogeneous background field induces spatially varying phase modulation between odd and even readouts, making conventional ghosting and distortion correction potentially unreliable depending on the degree of field inhomogeneity.

### 2.2 Eddy Currents From Nonlinear Gradient Switching

Eddy currents originating from rapid switching of diffusion sensitizing gradients cause imaging artifacts, including shearing, scaling, and position shifts [61]. The nonlinear gradient can be profiled by even‐order functions, as it exhibits even symmetry in *x*, *y* and *z*. This feature allows minimal interference with the first‐order linear gradient channels but induces strong coupling with the zeroth‐order main field coil [62], manifested as a slowly decaying Larmor frequency offset. Besides the eddy currents induced in the main field coil and linear gradient coils, there may also exist nonidealities in the nonlinear gradient coil waveform, either from eddy currents or imperfections in the amplifier chain. Previous studies reported preliminary results [24, 63]; when the nonlinear gradient coil is switched at the maximum slew rate, there is a long‐lasting Larmor frequency offset (i.e., a spatially uniform term) and a fast‐decaying component that mirrors the gradient shape.

Larmor frequency offset is expected to shift the object globally in the low‐bandwidth phase encoding direction of EPI. Consequently, aliasing artifacts will emerge from a mismatch between the pre‐calibrated coil sensitivity profile and the shifted image. A straightforward solution is to measure the exact Larmor frequency offset at the acquisition time and shift the acquisition frequency accordingly. The Larmor frequency offset is approximately constant during the short readout, in contrast to the timescale of the long decay.

Another effect of the induced Larmor frequency shift is that it can cause misalignment between the slices profiles of the excitation and refocusing pulses. Two approaches are to either shift the center frequency of the refocusing pulse to account for this or to use a wider bandwidth refocusing pulse. This work uses the latter strategy, which is common in many vendor sequences.

On the other hand, the spatially varying eddy currents from the nonlinear gradient itself (or other contributions that lead to imperfect waveforms) have only a minor effect on EPI readout. Their amplitude is negligible and decays within a few hundred microseconds under slow gradient switching conditions. In principle, it can also be minimized with appropriate pulse pre‐emphasis [64].

### 2.3 Mapping of the Nonlinear Field and Adjustment for EPI

The nonlinear field map, acquired by phase‐incremented Gradient Recalled Echo (GRE) imaging [65], was previously proven to be consistent across repeated installations and removals from the scanner bore. Therefore, there is no need for nonlinear field mapping at every scan session [25]. The GRE field map acquired with a high readout bandwidth is not sensitive to distortion.

EPI, however, is susceptible to distortion by an inhomogeneous static background field, leading to a geometric mismatch between acquired EPI‐DWI and the GRE field map. Because field inhomogeneity increases with proximity to the coil, the extent of distortion depends on the prostate slice position, which is subject‐specific.

Conventional correction methods, e.g., acquiring a pair of images with flipped phase encoding [55], might fail to deliver expected results due to overly large distortion. Therefore, a workaround is to retrospectively distort the measured nonlinear gradient map according to the static background field. Practically, we acquired the background field for each subject, and the nonlinear gradient map measured by GRE is retrospectively distorted only in the region of the prostate.

### 2.4 Diffusion Encoding Scheme With the Nonlinear Gradient Coil

The general definition of the *b*‐value for DWI [66] is

(1) $$b\left(x,y,z\right)=\int_{0}^{\tau }q^{2}\left(x,y,z,t\right)\mathrm{dt}$$

where

(2) $$q\left(x,y,z,t\right)=\gamma \int_{0}^{t}G\left(x,y,z,t^{\prime }\right)dt^{\prime }\begin{array}{c} =\gamma \int_{0}^{t}\left[G_{L}\left(t^{\prime }\right)+G_{\mathrm{NL}}\left(x,y,z,t^{\prime }\right)\right]dt^{\prime } \end{array}$$

Vectors $G_{L}$ and $G_{\mathrm{NL}}$ stand for linear and nonlinear gradients, $G=G_{L}+G_{\mathrm{NL}}$.

Although ramping the nonlinear gradient coil at its maximum slew rate maximizes the gradient moment and yields the highest possible *b*‐value, in this work we align its ramp and plateau with the linear counterpart for two reasons: (1) it drastically simplifies the *b*‐value calculation; (2) the linear gradient coils of the commercial MRI scanners have conservative switching rates, and having the nonlinear gradient coil following the same waveform profile reduces the problematic eddy currents that mirror the shape of nonlinear gradient. These benefits were deemed to outweigh the slight loss of *b*‐value associated with slower ramping.

Therefore, in the case of pulsed‐gradient spin‐echo EPI, the *b*‐value is formulated as [66]

(3) $$\begin{array}{cc} b\left(x,y,z\right) & =\gamma^{2}G{\left(x,y,z\right)}^{2}\left[\delta^{2}\left(∆-\frac{\delta }{3}\right)+\frac{1}{30}\epsilon^{3}-\frac{1}{6}\delta \epsilon^{2}\right]\\ & =\gamma^{2}{\left[G_{L}+G_{\mathrm{NL}}\left(x,y,z\right)\right]}^{2}\left[\delta^{2}\left(∆-\frac{\delta }{3}\right)+\frac{1}{30}\epsilon^{3}-\frac{1}{6}\delta \epsilon^{2}\right] \end{array}$$

where γ is the gyromagnetic ratio, ϵ,δ,Δ are the ramp time, pulse duration, and pulse interval, respectively.

## 3 Methods

The nonlinear gradient coil (Tesla Engineering Ltd., Storrington, UK) was inserted into a 3 T MAGNETOM Prisma (Siemens Healthineers AG, Erlangen, Germany), where the maximum linear gradient strength is 80 mT/m.

Initial studies were performed in phantoms, followed by experiments in 6 healthy male subjects (48.3 ± 12.0 years old), and finally in 8 patients (70.5 ± 9.4 years old, 10 PI‐RADS ≥ 3 lesions in total; details provided in Table S1). Subjects provided written informed consent in accordance with IRB approval. All the patients had a recent MRI scan in conventional scanners (< 6 months), and the lesions were marked by radiologists. There was neither a recent biopsy (< 6 months) nor radiation treatment before the imaging session.

### 3.1 Static Background Field Characterization

Four axial slices with 3 mm thickness were selected for characterization of the static background field, which are 40, 48, 72, and 88 mm away from the coil surface, respectively. These slices represent typical locations of the prostate observed in our study, given that the prostate has an average size [67] of 3 × 4 × 2 cm^3^ and is typically larger in senior men who need MRI screening.

The background field mapping protocol was 2D GRE: resolution 1.5 × 1.5 mm^2^, Field of View (FOV) 240 × 240 mm^2^, TR = 50 ms, flip angle = 15°, bandwidth = 1000 Hz/pixel. The background field was derived from the phase difference of images at TE = 11 ms and 12 ms.

To examine susceptibility artifacts, we acquired single‐shot EPI prostate images with phase‐encoding acceleration on healthy subjects at the above‐mentioned slices with the following parameters: resolution 1.5 × 1.5 mm^2^, FOV 240 × 240 mm^2^, TR/TE = 2500/42 ms, bandwidth = 1358 Hz/pixel, GRAPPA = 3, partial Fourier = 6/8, 3 averages, fat suppression with SPectral Attenuated Inversion Recovery (SPAIR). The same experiment was repeated for a phantom to examine ghosting artifact correction using 1D LPC, quantified by ghost‐to‐signal ratio [68].

For a more comprehensive analysis, we repeated the experiment on the left side of the coil using a representative sagittal leg slice, shown in Figure S1. In addition, the GRAPPA factor was chosen to balance distortion and SNR, i.e., the lowest GRAPPA factor that produced minimal distortion. The effect of GRAPPA factor was also evaluated on the sagittal leg slice and is shown in Figure S1.

### 3.2 Eddy Currents Characterization

A repeated GRE sequence was developed for the characterization of eddy currents. At each phase encoding step, two trapezoidal pulses of nonlinear gradient were played out to mimic the diffusion sensitizing gradient pulse, followed by repeated RF pulses with a regular 10 ms interval that records the phase evolution. The schematic diagram is shown in Figure S2. This eddy current mapping protocol was tested with an axial slice in the front (40 mm away from the coil surface) on a water phantom, and the acquisition parameters were the same as above except for TE = 5 ms, TR = 10 ms, repetition number = 1000. It was also repeated with a coronal slice for through‐slice eddy current mapping, shown in Figure S3. There was a one‐second period of blank time before the nonlinear gradient pulse to reduce interference from the last pulse. The dynamic eddy current can thus be characterized at TR intervals, for a duration up to TR × repetitions. The dynamic eddy current map was derived from the phase difference between a pair of scans: one with a nonlinear gradient pulse and one without.

We performed principal component analysis on the eddy current map ω(x,y,t) by singular value decomposition (SVD) in MATLAB (The MathWorks, Natick, MA, USA):

(4) $$\begin{array}{c} \omega \left(x,y,t\right)=\sum_{i=1}^{N}U_{i}\left(x,y\right)\cdot \mathrm{diag}\left(S_{i}\right)\cdot V_{i}{\left(t\right)}^{T} \end{array}$$

where the left singular vectors $U_{i}\left(x,y\right)$ represent spatial components, and the right singular vectors $V_{i}\left(t\right)$ correspond to the associated temporal trajectories, scaled by the singular values $\mathrm{diag}\left(S_{i}\right)$.

This experiment was repeated for multiple waveforms with varying amplitudes and durations. The Larmor frequency at the center of the EPI readout was linearly fitted to the waveforms' amplitudes and durations, and the resulting models can be used to predict the Larmor frequency for any given waveform.

### 3.3 Mapping of the Nonlinear Field for *b*‐Value

The volumetric field map (resolution 1.5 × 1.5 × 2 mm^3^, FOV 240 × 240 × 144 mm^3^) of the nonlinear gradient was acquired with a water phantom using GRE field mapping [65, 69], and the gradient was derived by spatial differentiation of the field map. A Gaussian filter (σ = 2) was applied to the gradient map to ensure spatial smoothness in the face of noise. The nonlinear gradient map has been proven reproducible across measurements, so it was used for all subjects [25]. It was retrospectively distorted in the region of the prostate to match the actual prostate in EPI [70], where the pre‐acquired background field map determined the extent of distortion.

### 3.4 Subject Positioning

The subject positioning steps are (1) initial setup of the nonlinear gradient coil at the edge of scanner bore, (2) temporary placement of the subject to securely bring the pelvis close to the coil in feet‐first orientation, and (3) moving the subject and coil to the isocenter simultaneously, and (4) fixing the coil to the rail with bolts (Figure 1f–h). It is bolted at a position that would put the prostate approximately at the isocenter. An 18‐channel flex receive coil is placed on the subject and a 4‐channel flex coil underneath.

### 3.5 Prostate DWI Acquisition

**FIGURE 2:**
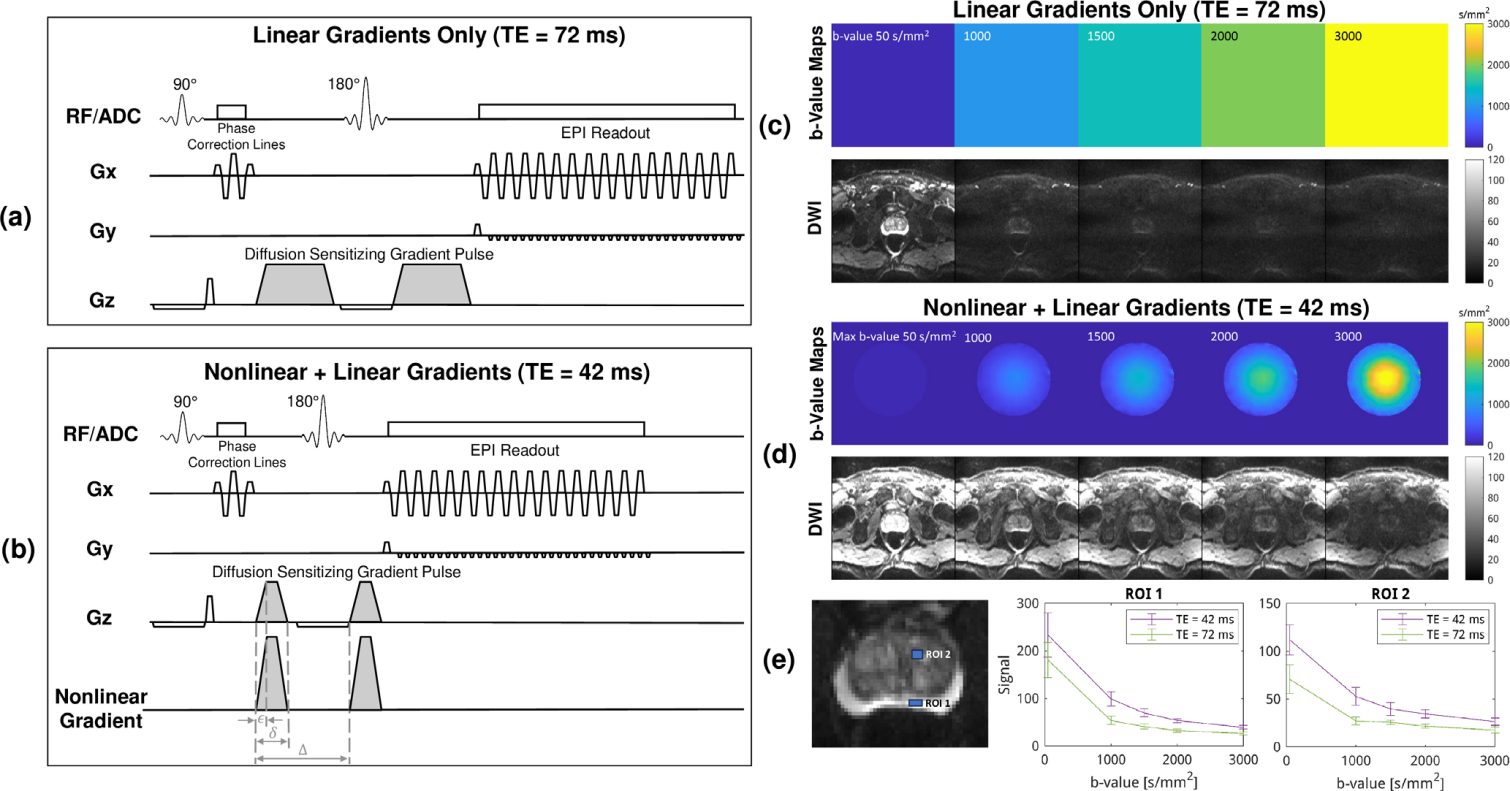
PGSE single‐shot DWI was used as the pulse sequence for prostate DWI in this study. At a fixed *b*‐value, TE was reduced from 72 ms (a) to 42 ms (b) by the strong gradient amplitude of the nonlinear gradient coil. The nonlinear gradient waveform shares the same ramp time (*ϵ*), duration (*δ*), and interval (Δ) as the *z*‐axis linear gradient channel. Signal decay induced by the linear diffusion‐encoding gradients (c) is spatially homogeneous, whereas that induced by the combined nonlinear and linear gradients (d) is spatially variant, even though the *b*‐values in the prostate region are identical. The shorter TE enabled by the nonlinear gradient results in higher signal intensity across the field of view, and the signal was plotted against *b*‐values (e) for two regions of interest in the peripheral zone and the transition zone.

DWI was acquired with pulsed gradient spin echo (PGSE) single‐shot EPI: resolution 1.5 × 1.5 × 3 mm^3^, FOV 240 × 240 mm^2^, anterior–posterior phase encoding, TR = 2500 ms, bandwidth = 1358 Hz/pixel, GRAPPA = 3, partial Fourier = 6/8, 12 repetitions, fat suppression with SPAIR. The nonlinear gradient waveform had the same ramp time (ϵ), duration (δ), and interval (Δ) as the linear gradient waveform, shown in Figure 2.

Because gradient strength varies across slices, we implemented an on‐the‐fly optimization of TE for the prostate slice of interest. Given the maximum gradient strength available at a specific slice, we calculated the minimum TE required to satisfy the target *b*‐values. The gradient waveform amplitude was then scaled within the timing constraints to achieve the precise target *b*‐values.

Note that multi‐slice acquisition is feasible, but it would require different amplitudes of nonlinear gradient for each slice because the gradient strength varies significantly across axial slices. For proof‐of‐concept purposes, single‐slice acquisition was adopted in this work. Among all the subjects, the acquired slices are 30–76 mm away from the nonlinear gradient coil surface.

With the above‐mentioned common parameters, two protocols were developed and tested on both healthy subjects and patients.

Protocol 1 has clinically typical moderate *b*‐values = [50, 600, 800, 1000] s/mm^2^ for ADC validation, where TE = 57 ms for linear gradients and 42–47 ms for nonlinear + linear gradients.

Protocol 2 has higher *b*‐values = [50, 1000, 1500, 2000, 3000] s/mm^2^ to suppress normal tissues and highlight lesions, where TE = 72 ms for linear gradients and 42–54 ms for nonlinear + linear gradients.

An additional set of 12 repeated *b* = 0 s/mm^2^ images was acquired at each TE for SNR calculation.

To demonstrate the SNR boost on scanners with lower gradient strength, we adjusted the linear gradient amplitude to 40 and 60 mT/m, in addition to the current 80 mT/m, for the scanning of one subject. We then designed protocols with TE that support *b*‐values up to 3000 s/mm^2^. For linear gradients, the TE values were 95, 78, and 72 ms, respectively. When combining both linear and nonlinear gradients, TE was reduced to 58, 56, and 54 ms, where the nonlinear gradient strength of the prostate slice was around 200 mT/m.

The spherical shape of prostate cells suggests almost isotropic diffusion, and we used a single‐directional scheme [71] by applying a linear diffusion sensitizing gradient pulse in the *z*‐axis, which is approximately the nonlinear gradient direction.

We input the actual waveform to the fitted functions of Larmor frequency offset (Section 3.2) to get the correct frequency on the fly without ad‐hoc measurement. It was then applied to the analog‐to‐digital converter during readout.

As a high‐resolution structural reference, Turbo Spin Echo *T*_2_‐weighted images were acquired at the same slices: resolution 0.94 × 0.94 × 3 mm^3^, FOV 240 × 240 mm^2^, TR = 8700 ms, bandwidth = 199 Hz/pixel, GRAPPA = 2.

### 3.6 Image Processing and Analysis

All images were processed retrospectively by denoising [72] and removing Gibbs ringing [73] (MRtrix3 v3.0.2 [74]). As the data was single‐slice, the third dimension of input data was arranged to represent *b*‐values, with the fourth dimension assigned to repetitions. Unaveraged DWI with and without denoising & deringing are shown in Figure S4. For SNR comparison, we calculated the SNR both before and after the processing.

The prostate masks were delineated on DWIs with *b*‐value = 1000 s/mm^2^. The region of interest (ROI) of the lesions was created according to radiologists' reports, and the ROI of normal tissues was selected on the contralateral side, if the lesion is small, or a neighboring region, if the lesion crosses the midline [7].

With Protocol 1, we calculated the *b*‐value map according to Equation (3) and the ADC map with the nonlinear gradient. We compared it to the linear gradient reference, quantifying it by normalized root mean square error (NRMSE) of the prostate ROI.

We calculated SNR maps at different TEs from *b*‐value = 0 s/mm^2^ images of all patients and healthy subjects. Note that we did not calculate SNR on images with diffusion weighting to avoid bias from spatially variant signal decay induced by the nonlinear gradient. SNR is defined by the mean value divided by the standard deviation across these repetitions (Equation (A5) in reference [75]).

For the calculation of the contrast‐to‐noise ratio (CNR) of high‐*b*‐value DWIs acquired in Protocol 2 for all patients, each nonlinear‐gradient DWI was first normalized by monoexponentially extrapolating its signal amplitude to the uniform *b*‐value of the corresponding linear‐gradient DWI (details shown in Figure S5). Due to the limited size of the prostate, *b*‐value variation within the gland was minimal, resulting in negligible changes in DWI signal amplitude after normalization. CNR at each *b*‐value was defined as the absolute difference between the mean signal intensities of the lesion and normal‐tissue ROIs, divided by the noise floor. The standard deviation of each pixel was calculated across repeated measurements of the *b* = 0 s/mm^2^ images, and the mean standard deviation within the prostate mask was defined as the noise floor. This reflects the practical noise floor for a clinical read, though it is higher than the system noise. Noise floor calculation based on unoccupied regions in the FOV was avoided as it was confounded by residual ghosting and aliasing.

RSI is a multicompartmental diffusion model that does not require varying diffusion times and can be directly applied to data acquired with Protocol 2. Recent studies have identified an optimal four‐compartment model using fixed, pre‐determined compartmental ADC values [36, 76]. Tumors are predominantly highlighted in the first compartment, *C*_1_, which represents restricted diffusion and has been shown to provide greater lesion conspicuity than conventional ADC [76, 77]. Lesion conspicuity was quantified on the *C*_1_ map using the lesion‐to‐normal‐tissue contrast ratio: $\left(C_{1\text{lesion}}-C_{1\text{normal}}\right)/C_{1\text{normal}}$.

We conducted a Wilcoxon signed rank test [78] for long‐TE DWI with linear gradients vs. short‐TE DWI with nonlinear + linear gradients, regarding SNR, CNR, and contrast ratio in *C*_1_. A *p*‐value of less than 0.05 was considered statistically significant. All analyses were performed using MATLAB.

## 4 Results

Figure 2a,b shows the pulse sequence of the DWI used in this study with and without the nonlinear gradient coil, and Figure 2c,d is a composite of example DWIs at equivalent *b*‐values. In the conventional case, the linear gradients created uniform diffusion weighting across the FOV with a long TE and lower brightness. Enhanced by the nonlinear gradient, DWIs had nonuniform diffusion weighting but achieved the same *b*‐values at the prostate with a short TE and higher brightness, confirmed by the signal plot from two ROIs in Figure 2e. Note that the outer regions experienced very low *b*‐values when using the nonlinear gradient and therefore did not exhibit substantial signal attenuation.

**FIGURE 3:**
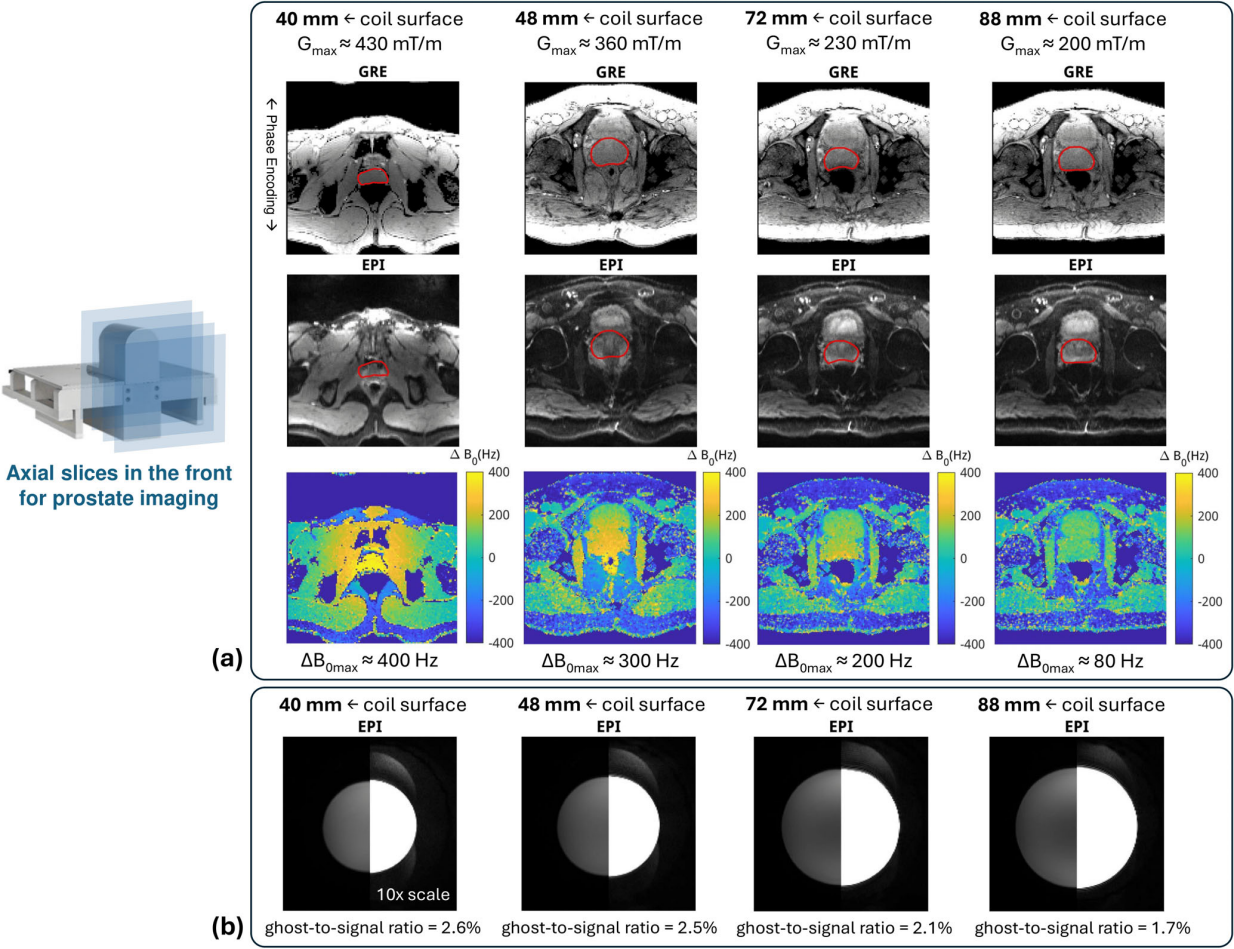
Background Δ*B*_0_ map, distortion, and ghosts. A static field inhomogeneity is introduced by placing the nonlinear gradient coil in the scanner bore. Four axial slices are shown, located 40, 70, and 100 mm from the coil surface. (a) GRE serves as reference, and the distortion in EPI corresponds to the measured Δ*B*_0_ map. Slices closer to the coil show greater distortion from increased B_0_ inhomogeneity, resulting in a significant mismatch of prostate contours between GRE and EPI. (b) The N/6 ghost is the most significant ghosting artifact (3× in‐plane acceleration, 2× EPI readout polarity). Ghosts of the phantom are not significant, as ghost‐to‐signal ratio < 3%.

Figure 3a depicts the background field induced by the nonlinear gradient coil in the scanner bore, along with the distortion it induces. The susceptibility artifact varied with slice position: slices farther away from the coil showed less prostate distortion, judging from the prostate contours in EPI and the corresponding GRE reference. This is expected with increasing distance from the copper insert coil. In fact, the farthest slice had negligible distortion. The susceptibility artifact aligned with the measured background field map (Δ *B*_0max_ from 400 to 80 Hz across slices). Figure 3b shows the ghosting artifact at the same slices. The N/6 ghost was the main component, resulting from a combination of 3× acceleration and odd/even readouts. As expected, ghosts also depended on the slice position. The largest ghost‐to‐signal ratio was less than 3%, suggesting insignificant ghosting artifacts. Although N/6 ghosts appeared in the phantom images, they were not visible in the in vivo prostate images; therefore, no additional effort was required for ghost correction. This difference was because the phantom had clear edges that enhanced the visibility of ghosting artifacts, whereas the complex body anatomy caused the ghost signal to disperse and appear more noise‐like.

**FIGURE 4:**
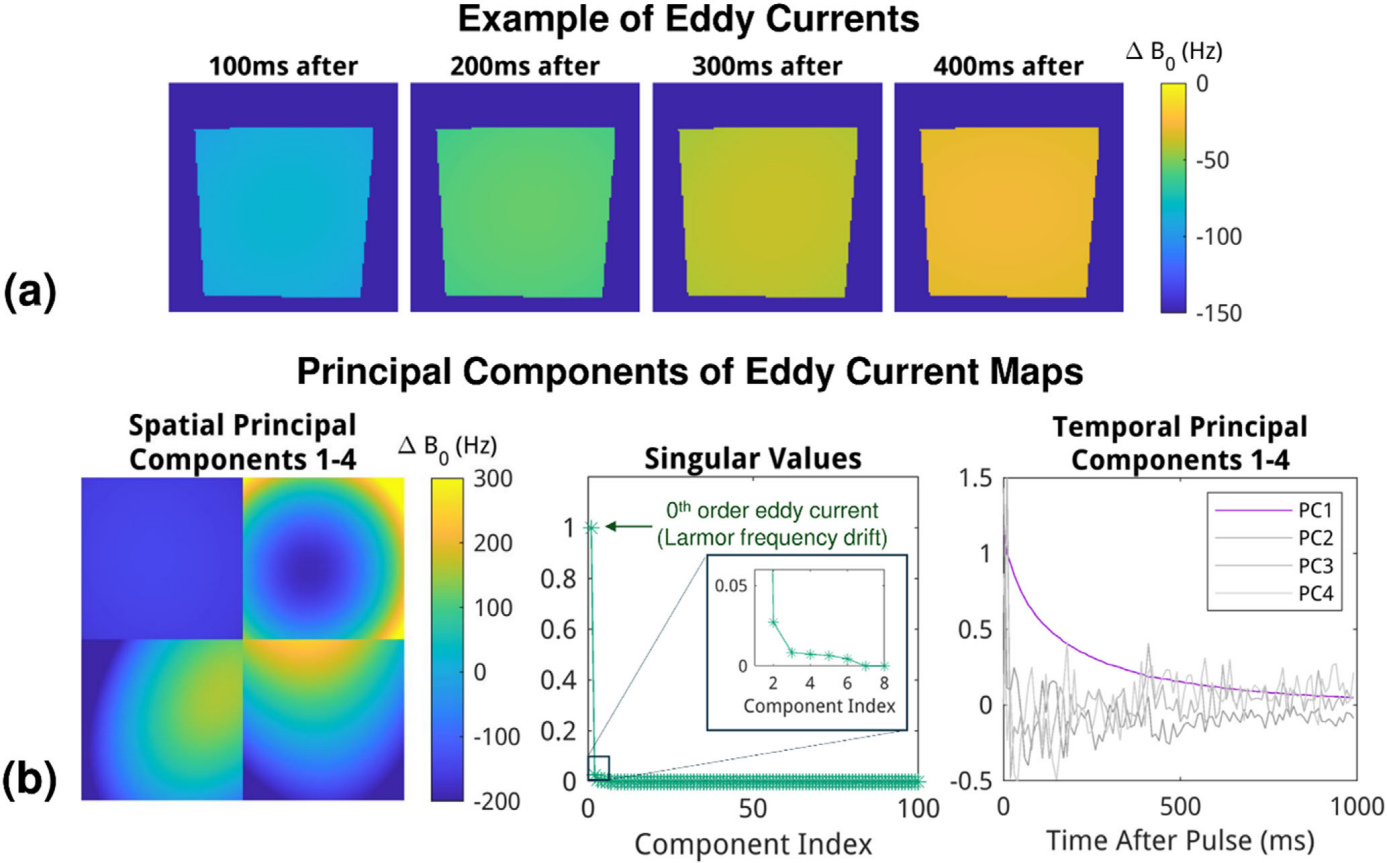
(a) Selected time frames of eddy current maps. Eddy currents are homogeneous across the imaging phantom, indicating a time‐dependent Larmor frequency offset. (b) Singular values from principal component analysis indicate that the dominant component is Larmor frequency offset, with remaining components representing minor higher‐order variations.

Figure 4a displays examples of eddy current maps of the nonlinear gradient coil. These maps reflected a spatially flat Δ *B*_0_ at each timepoint, indicating that a time‐varying Larmor frequency offset (zeroth‐ordered eddy current) dominates eddy currents. The principal component analysis in Figure 4b further supports that the first principal component, i.e., Larmor frequency offset, outweighs the other contributions. Note that the gradient was switched much more slowly than the maximum slew rate, using the same ramp time as the linear gradients that did not exceed the safety limit. Consequently, even though the remaining spatial components might include spatially varying fields, their amplitude was negligible after the initial fast decay of around 300 μs.

**FIGURE 5:**
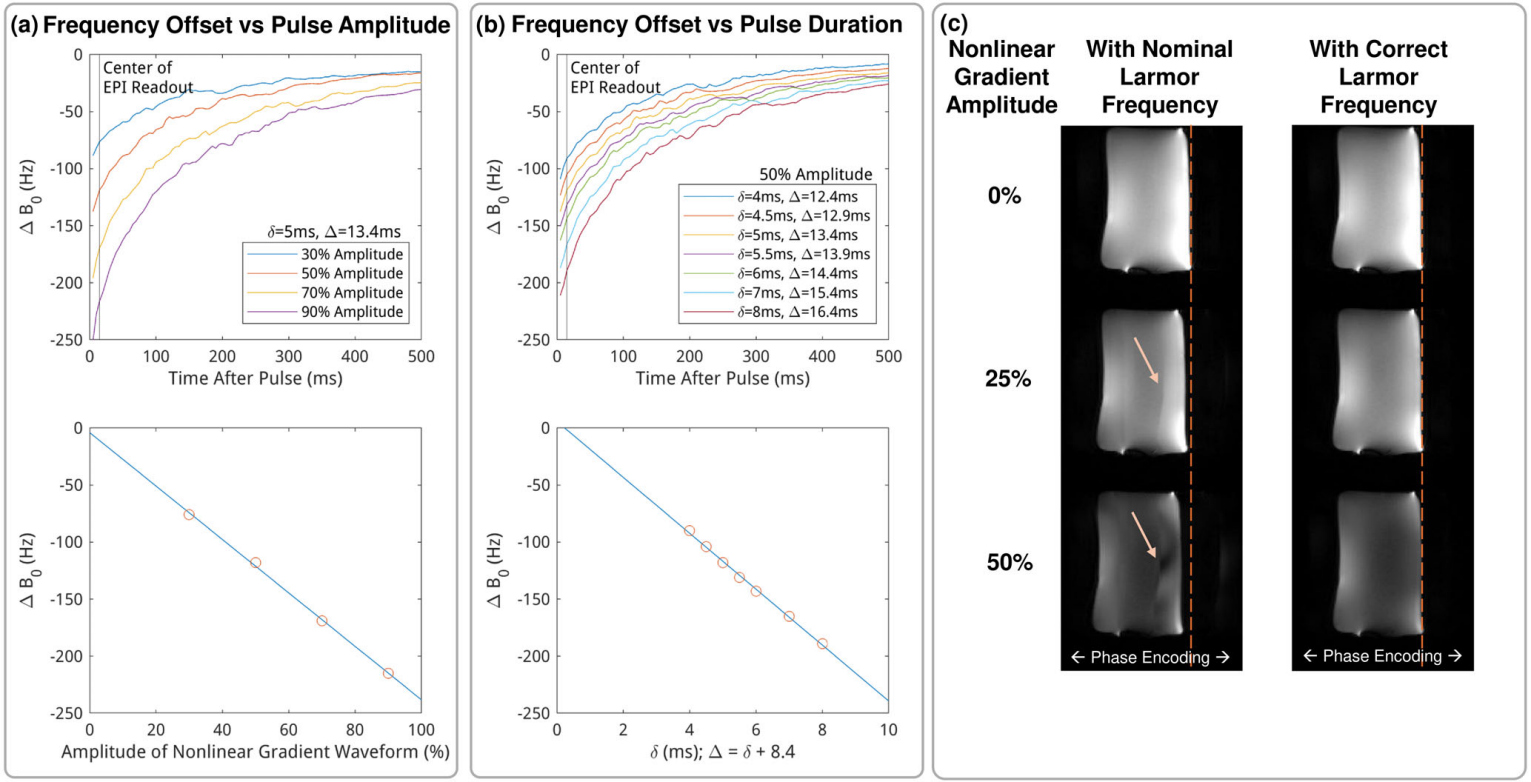
(a) Larmor frequency offset plotted over time for multiple waveform amplitudes; the frequency at the center of the EPI readout was extracted and is shown to scale with amplitude. (b) Larmor frequency offset for multiple waveform durations, shown to scale with duration. (c) Larmor frequency offset induces image shifts in the phase‐encoding direction and aliasing artifacts, which can be corrected by providing the correct frequency to the analog‐to‐digital converter.

Figure 5a,b further shows the relationship of Larmor frequency offset with respect to the diffusion waveform amplitude and duration. The Larmor frequency offset spanned on the order of seconds, shown by the upper subplots, and the long eddy current time constant made the frequency offset proportional to the duration [79]; the eddy current was also proportional to the waveform amplitude. The linear relationship helps predict the frequency offset needed for the analog‐to‐digital converter, and it obviates careful measurement of eddy currents for a new waveform. Note that with TR = 2.5 s, the Larmor frequency has returned to its nominal value by the time the next excitation pulse begins. Figure 5c demonstrates the correction of Larmor frequency offset for DWI. The global shift increases linearly with gradient amplitude. The correct frequency updated to the analog‐to‐digital converter shifted the imaging phantom back to the correct position. This correction also removed aliasing artifacts that would occur from a spatial shift between the image FOV and coil sensitivity profile.

**FIGURE 6:**
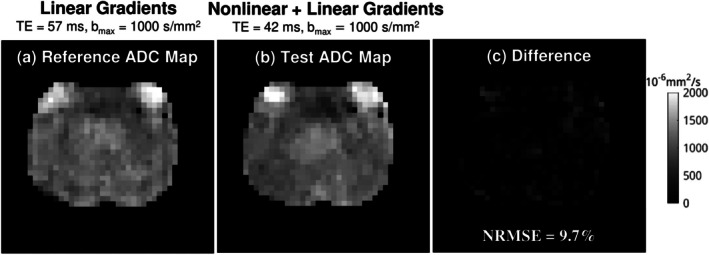
Using data acquired from Protocol 1 (*b*_max_ = 1000 s/mm^2^), the ADC of a healthy subject was calculated from (a) linear gradients with a long TE and (b) the combination of nonlinear and linear gradients with a short TE. The nonlinear gradient achieves the same *b*‐value at TE = 42 ms as the linear gradient reference at TE = 57 ms. The resulting ADC map (b) closely matches the reference map (a), with the corresponding difference map (c) showing only minor variations.

Figure 6 shows the ADC map of a healthy subject acquired using Protocol 1, demonstrating the feasibility and accuracy of ADC estimation with a nonlinear gradient–based *b*‐value map. The ADC map obtained with linear gradients, at TE = 57 ms and *b*_max_ = 1000 s/mm^2^, served as the reference. The ADC derived from combined nonlinear and linear gradients, acquired at a shorter TE of 42 ms but with the same *b*_max_ = 1000 s/mm^2^, closely matched the reference (NRMSE = 9.7%). This level of deviation is consistent with short‐term measurement variability [80] and with ADC‐*T*_2_ correlation arising from multiple microscopic compartments (stroma, epithelium, and lumen) [41, 42, 43]. On the other hand, attempting to acquire DWI with linear gradients at TE = 42 ms would allow for *b*_max_ = 50 s/mm^2^, which is insufficient for accurate ADC estimation for most biological tissues.

**FIGURE 7:**
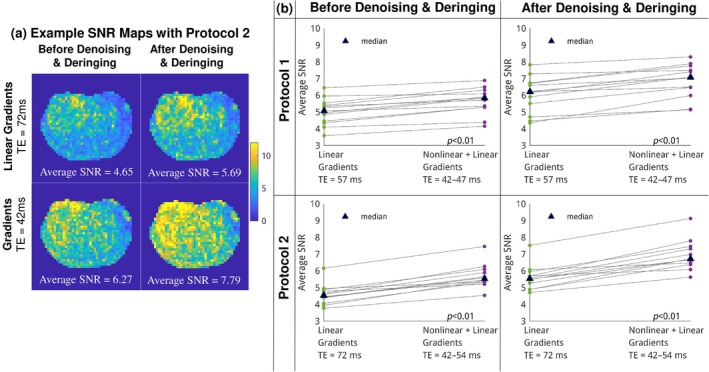
(a) SNR maps of an example subject acquired with Protocol 2. (b) Paired plots comparing SNR. Each dot represents the average SNR of a subject. Protocol 2 (TE 72 vs. 42–54 ms) demonstrates a greater SNR advantage from the nonlinear gradient than Protocol 1 (TE 57 vs. 42–47 ms).

Figure 7a shows example SNR maps of a healthy subject with Protocol 2, and the average SNR was 6.27 vs. 4.65 (before image processing), and 7.79 vs. 5.69 (after image processing). Figure 7b compares the average prostate SNR, with each dot representing a subject. The nonlinear gradient boosted SNR both before and after denoising & deringing, in both Protocol 1 and Protocol 2. Protocol 2 witnessed a larger SNR boost as expected from the larger TE difference. Quantitatively, after standard practice denoising and deringing, SNR of DWI was boosted by 3%–38% (median 16%, *p* < 0.01) for Protocol 1, and 7%–38% (median 26%, *p* < 0.01) for Protocol 2. This level of SNR boost is consistent with the TE reduction and typical prostate *T*_2_ value in average [81].

**FIGURE 8:**
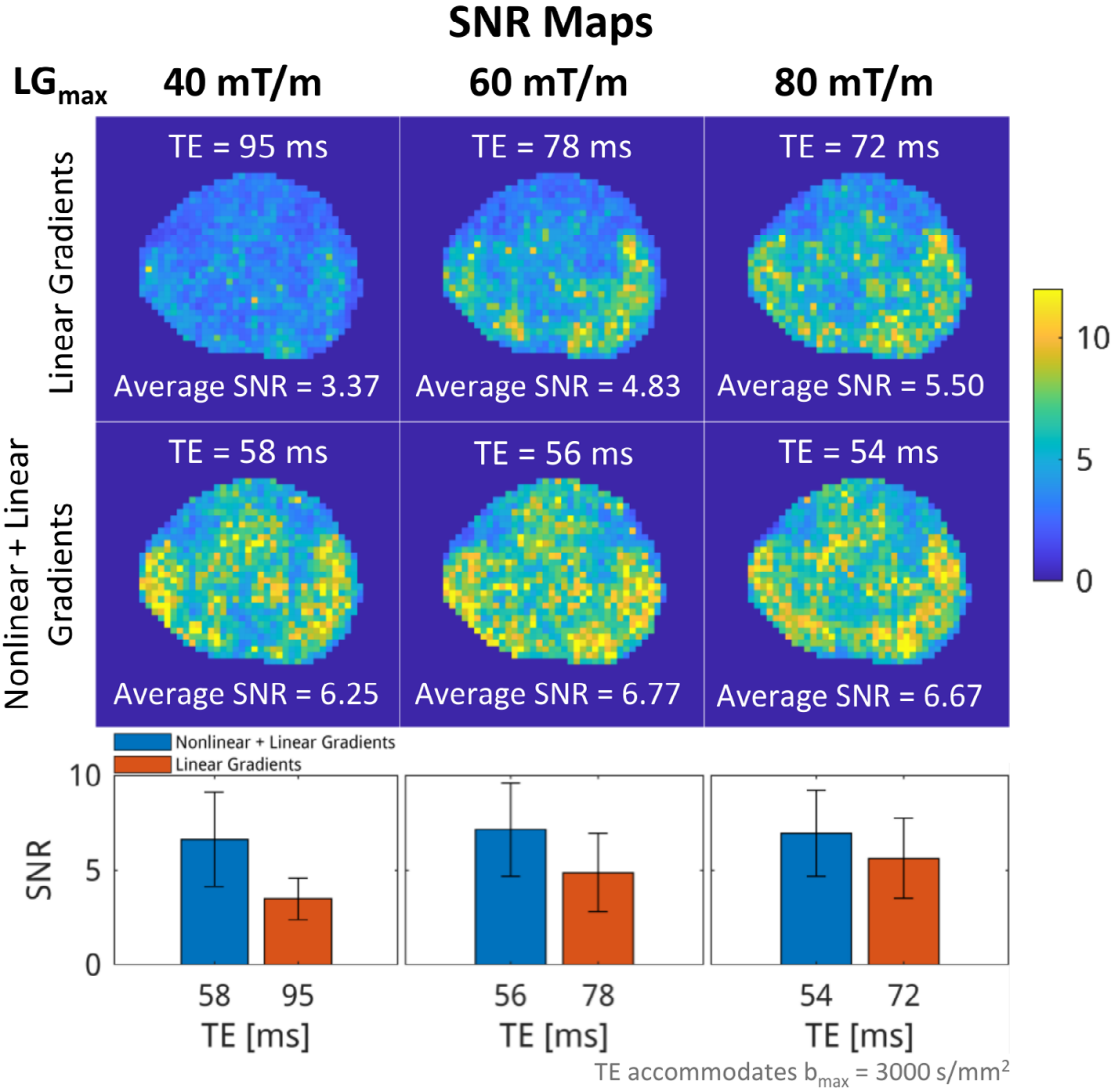
SNR maps were acquired using maximum linear gradient amplitudes of 40, 60, and 80 mT/m. In each case, TE was sufficient to accommodate a diffusion gradient waveform for *b*_max_ = 3000 s/mm^2^. The minimum achievable TE is substantially shorter with the combined nonlinear and linear gradients than with linear gradients alone at the same diffusion weighting. As the TE advantage increases, the SNR gain becomes more pronounced, highlighting the benefit of the insert gradient coil on scanners with limited gradient strength.

Figure 8 demonstrates further SNR boosts in common scanners with weaker gradient strength, i.e., 40 and 60 mT/m. TE that accommodated a maximum *b*‐value of 3000 s/mm^2^ yielded larger reduction with weaker gradient strength, corroborated by more significant SNR improvements, i.e., 6.25 vs. 3.37 at 40 mT/m, 6.77 vs. 4.83 at 60 mT/m, and 6.67 vs. 5.50 at 80 mT/m.

**FIGURE 9:**
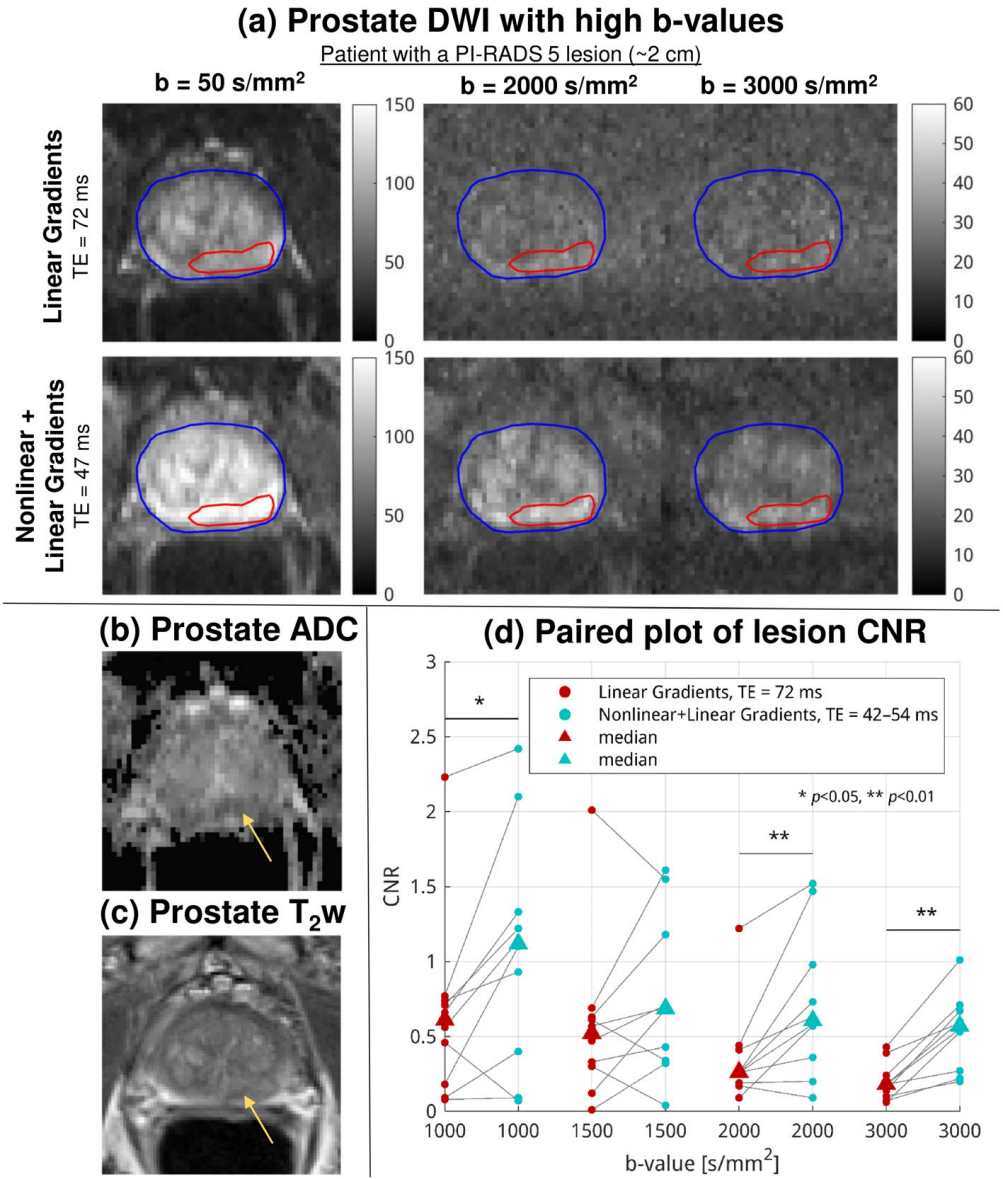
(a) Prostate DWIs acquired using Protocol 2 with high *b*‐values. The lesion (red contour) is not visible above the noise at *b* = 3000 s/mm^2^ DWI with linear gradients only (TE = 72 ms), but conspicuity is greatly increased at the same *b*‐value with combined nonlinear and linear gradients (TE = 47 ms), showing higher CNR. The lesion is confirmed by the ADC map (b) and the *T*_2_w image (c), although contrast in the *T*_2_w image is suboptimal. (d) Paired plot of CNR for individual lesions across *b*‐values from 1000 to 3000 s/mm^2^ in Protocol 2. The use of nonlinear gradients enhances CNR at all *b*‐values, with statistically significant improvements at higher *b*‐values.

Figure 9a shows example high‐*b*‐value DWIs of a patient with a PI‐RADS 5 lesion in the peripheral zone, acquired with Protocol 2. Protocols with only linear gradients (TE = 72 ms) produced considerably lower signals than those with nonlinear + linear gradients (TE = 47 ms). The lesion, located in the left posterior peripheral zone and measuring approximately 2 cm at its greatest dimension, was almost invisible on high‐*b*‐value DWIs acquired with only linear gradients but remained clearly visible with nonlinear gradients. The lesion was further confirmed by the conventional ADC map Figure 9b (linear gradient, *b*_max_ = 1000 s/mm^2^) and the *T*_2_w image (Figure 9c), although the *T*_2_w image showed poor lesion contrast.

Figure 9d depicts the boost of lesion CNR via a paired plot of the lesion CNR for all patients. The nonlinear gradient reduced TE from 72 to 42–54 ms, so the remaining signal in the lesion was preserved from *T*_2_ decay. This was shown across all the *b*‐values from 1000 to 3000 s/mm^2^ in Protocol 2. The CNR was mostly improved with reduced TE. In high‐*b*‐value DWIs, CNR increased uniformly and significantly (*p* < 0.01), with a median value of 133% at *b* = 2000 s/mm^2^ and 217% at *b* = 3000 s/mm^2^.

**FIGURE 10:**
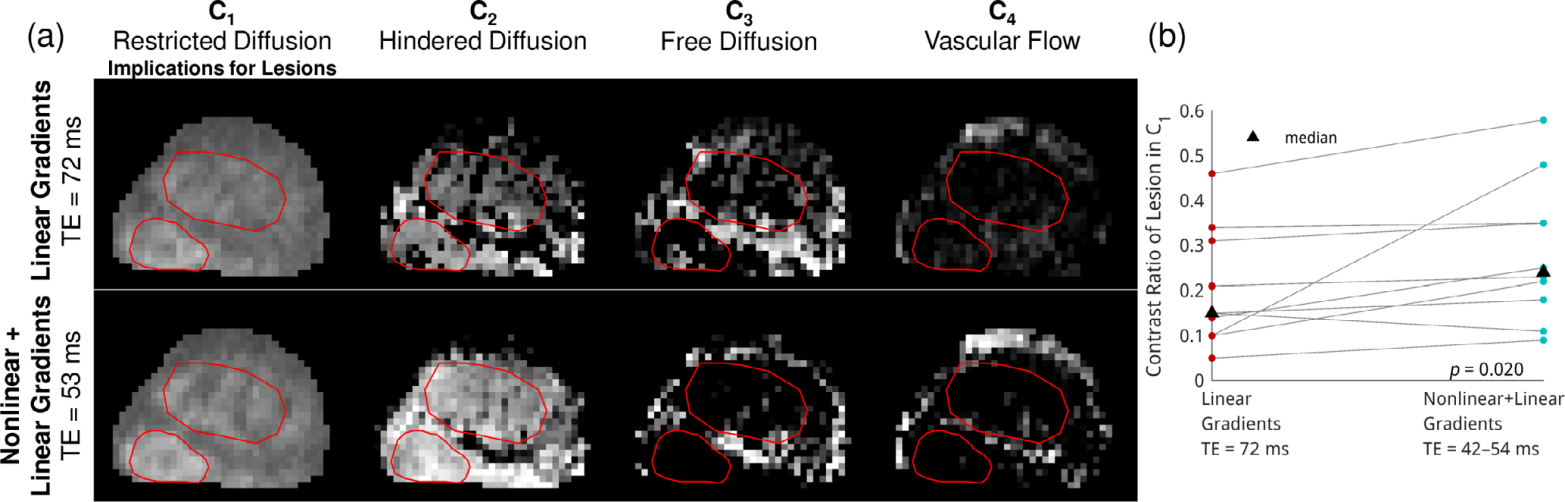
(a) Example compartmental diffusivity maps of a patient with two PI‐RADS 5 lesions, acquired with a long TE (72 ms) and a short TE (53 ms, enhanced by nonlinear gradients). The lesions show high signal in the *C*_1_ map, associated with restricted diffusion, and the short TE further accentuates this restricted diffusion. Hindered diffusion also becomes more apparent at short TE. (b) Paired plot showing consistent improvement in the lesion‐to‐normal tissue contrast ratio in the *C*_1_ map across all patients. The improvement with the short TE is statistically significant (*p* = 0.02).

Figure 10a shows a representative series of compartmental diffusivity maps from a patient with two PI‐RADS 5 lesions, derived from the RSI model fitted to data acquired using Protocol 2. The lesions exhibited a greater proportion of signal in the *C*_1_ map than the normal tissue, suggesting increased restricted diffusion components, which are associated with a higher epithelial cell partial volume. Notably, the *C*_2_ map, which reflects hindered diffusion from tortuous extracellular spaces, gains detectability at reduced TE, since this component has a short *T*_2_ similar to that of the *C*_1_ map, both being related to a high epithelial cell volume. Figure 10b presents the contrast ratio of lesion to normal tissue in the C_1_ map for all the 10 lesions from the enrolled patients. This demonstrated a significant improvement (median 23%, *p* = 0.02) in lesion conspicuity with short TE.

## 5 Discussion

In this study, we first analyzed the imaging performance of an inside‐out nonlinear gradient coil for DWI and proposed necessary artifact correction. We then developed imaging protocols specialized for prostate DWI exploiting the locally strong gradient (200–500 mT/m). These included moderate‐*b*‐value DWI in routine clinical use to derive ADC maps and high‐*b*‐value DWI to highlight the lesion. The latter protocol allows for a drastically reduced TE and consequently a higher signal level. The nonlinear gradient map creates ADC maps with high fidelity. Furthermore, we quantified improved SNR at the prostate, enhanced lesion‐to‐normal‐tissue CNR, and greater visibility of restricted diffusion in lesion ROIs. Notably, the RSI analysis confirms that at high *b*‐values, long‐*T*_2_ luminal signals are largely suppressed, and image brightness is dominated by shorter‐*T*_2_ compartments crucial for cancer detection. It corroborates with the DWI result that the residual signal at high *b*‐values became significantly more visible at reduced TE. As a result, the shorter TE enabled by the nonlinear gradient coil leads to higher lesion contrast. On the other hand, the brightness of low‐*b*‐value DWI was dominated by the long‐*T*_2_ luminal compartment that gained little from reduced TE.

### 5.1 Artifacts

The proposed nonlinear gradient coil creates field distortion in the axial pelvis FOV that is not easily corrected with standard shimming. The strong gradient is not used for EPI readout, and thus distortion cannot be mitigated by a short readout train. Although slices far away from the coil are barely distorted, closer slices provide superior gradient strength, albeit at the cost of off‐resonance up to 400 Hz across the prostate. Instead of the conventional correction method [55], we intentionally distorted the nonlinear gradient map for the prostate to match its location in DWI. Slices further away, however, show only marginal distortion, so conventional correction methods would still work. In fact, this is often the case for patients who have difficulty positioning their pelvis close to the coil surface. As such, conventional distortion correction [55] will be incorporated in the next version of the protocol, with possible extension to Blip‐Up and ‐Down Acquisition [56] and deep neural networks [82].

With slow ramping, the zeroth‐ordered Larmor shift is the major eddy current because of the coupling of the symmetric nonlinear gradient coil and the zeroth‐ordered main field coil. The eddy currents from the linear gradients are minimal, and we did not observe shearing or scaling [61]. Other components were negligible in the context of this study.

In multi‐slice acquisition Larmor frequency drift would accumulate, which must be addressed before clinical deployment of this sequence. A negatively‐polarized pulse of nonlinear gradient after readout would compensate for the frequency offset between slices to reduce this accumulation. Multislice acquisition could also introduce crosstalk from the wider bandwidth refocusing pulse used here. An alternative approach would be to adjust the RF center frequency to follow the actual Larmor frequency.

The nonlinear gradient produces transverse fields (concomitant field) [83], which have been mapped in previous work [24, 84]. However, the transverse components are minimal for ROIs near the axis of the nonlinear gradient coil, which is the typical prostate position. The comparison is presented in Figure S6 using data from our previous report [24]. There is no visible difference between the nonlinear gradient maps of the total and longitudinal fields at the prostate location (40–90 mm from the coil surface), with discrepancies of less than 0.01 mT/m. This has negligible impact on *b*‐values, and any evolution induced by concomitant field is balanced by the spin echo. Any concomitant field terms would not impact spatial encoding because the nonlinear gradient is used only for diffusion encoding.

### 5.2 Well‐Being of Patients

As previously reported [24, 85] and further confirmed in this study, there has been no peripheral nerve stimulation or mechanical resonance reported, although there is vibration from the nonlinear gradient coil and the flat platform. Therefore, the coil will not harm the patients with nerve stimulation or unsafe movement even if it is not fixed to the rail.

To fully exploit the locally strong gradient, the prostate should be as close to the coil as possible, so we ensured the relative position at the entrance of the bore before sending them to the isocenter. This approach also avoids risk of the pelvis bumping into the coil and patient legs are supported throughout the positioning process.

Human subjects were provided scrotal support, typically underwear with a built‐in jock strap. Without this additional support, the testicles can fall between the gradient and the groin, which could cause discomfort and impede close proximity between the prostate and the coil.

The Prisma scanner used in this work has a 60 cm‐diameter bore, but a wide‐bore (∼70 cm) scanner would be preferable to make prostate screening available for a larger population group. In this work, accommodation of the legs around the gradient did not pose any challenges, but some larger patients could not be scanned due to discomfort with the narrow scanner bore at the shoulders.

### 5.3 Limitations

Limitations of this work include the use of clinical imaging resolution and a lack of gold standard validation. Visual inspection of some high gradient images seems to suggest observable heterogeneity within lesions, and the signal intensity at high *b*‐value may allow for smaller voxels. However, in the absence of ground‐truth histology, our analyses used the ROIs defined on standard DWI. ROIs based on histology, particularly in higher‐resolution scans, could provide data to explore potential relationships between DWI signals and Gleason scores in future studies.

To better align the slices with the organ, oblique axial slice is sometimes preferred for prostate DWI [30]. This approach is compatible with the nonlinear gradient coil; however, larger obliqueness increases the variation in gradient strength within prostate, resulting in a more nonuniform *b*‐value.

The locally strong gradient makes prostate lesions the most suitable application, whereas extra‐capsular lesions are exposed to slightly weaker gradient strength. In contrast, lymph node metastases are located very far from the coil surface, where the gradient strength becomes negligible, leaving little room for improvement.

The lack of directionality is a limitation, but it does not impede routine clinical evaluation of prostate cancer, which primarily relies on ADC and DWI signal intensity. The main pathological features of malignancy involve changes in the fraction of luminal, stromal, and epithelial compartments, which single‐directional DWI is sensitive to. However, this coil may be less suitable for models that require detection of diffusion anisotropy.

One additional limitation is that SNR may have been relatively low at the current spatial resolution, making it difficult to detect normal tissue at high‐*b*‐value DWI.

In the future, multi‐slice acquisition will necessitate varied waveform amplitudes across slices for uniform *b*‐values and constant TE across the volume, so the overall improvement would be limited by the gradient strength at the farthest slice. The minimum TE will differ by approximately ∼10 ms, depending on the location of the farthest prostate slice, as shown in this and previous studies [24]. Because the nonlinear gradient is played in tandem with linear gradients, there is always TE reduction, even if the entire volume is acquired at the longest TE for the furthest slice.

### 5.4 Prospects

The TE reduction achieved in this work is primarily benchmarked against linear gradients with a maximum amplitude of 80 mT/m. More significant SNR improvements are observed with 60 mT/m and 40 mT/m gradients. While state‐of‐the‐art clinical systems have increasingly adopted gradient systems of 80 mT/m [86], there are still many clinical scanners that operate with 40 mT/m gradients [5, 87]. From this standpoint, the nonlinear gradient coil offers an affordable and accessible solution to high‐performance prostate DWI without upgrading the whole‐body scanner. A weaker gradient strength and slew rate may increase the EPI readout time, but it is independent of TE reduction from the nonlinear gradient diffusion waveform. Moreover, partial Fourier is widely used, so the impact of weaker gradient strength and slew rate is smaller on TE than on the total readout time. A recent series of commercial mid‐field MRI scanners are equipped with a wider bore (≥ 80 cm) and relatively weak linear gradients (26 mT/m) [88], which may represent an ideal use case for a nonlinear gradient coil. As there is evidence in the literature that setting a lower prostate specific antigen (PSA) threshold for MRI could improve the number of clinically significant cancers detected without increasing overdiagnosis [89], better prostate DWI on commonly accessible scanners is desirable.

The installation and use of the nonlinear gradient coil are efficient and on demand, without interfering with routine clinical scans. The implementation pipeline can be fully automated in the future, such as determining gradient waveform duration and amplitude. Importantly, except for the subject‐dependent static background *B*_0_ mapping, if needed, no additional scan is needed per subject. The nonlinear gradient map can be obtained beforehand and has been shown to be reproducible [25].

To further reduce TE in the framework of single‐shot EPI, one can compress the diffusion sensitizing gradient waveform by exploiting the time before the refocusing pulse (EN‐CODE) [90] or before the excitation pulse (Pre‐ENCODE) [91], with additional benefits on suppressing eddy currents. This has been shown to benefit prostate DWI with high spatial resolution [92]. However, the TE reduction may be marginal since the monopolar diffusion waveform has already been compressed with the nonlinear gradient coil. Another possibility to improve SNR would be to combine nonlinear gradient encoding with a more efficient readout strategy. Spiral trajectories [93] allow a minimal TE at the beginning of readout and have been applied with a dynamic field‐camera [8, 94].

This work used a SE‐EPI DWI sequence, which is the typical clinical approach to prostate DWI. Given the shorter gradient pulses needed, other approaches merit further consideration, e.g., gradient echo or steady state free precession (SSFP) sequences, so further gains may be possible.

Time‐dependent diffusion for microstructural characterization of prostate with the nonlinear gradient coil is feasible [40]. Probing short‐time‐limit microstructure provides direct access to small length scale but often requires oscillating gradient spin‐echo (OGSE) [95]. However, the limited gradient amplitude of commercial scanners necessitates prolonged TE for OGSE of sufficient *b*‐value, which renders poor SNR. Its application to human imaging has been limited [87, 96]. Recently, strong gradients available in head‐only scanners have been leveraged for brain microstructural imaging with OGSE [97, 98, 99].

Skeletomuscular microstructural imaging is also possible with this coil by placing legs on the side of the coil, but probing short diffusion time is even more challenging due to significantly lower *T*_2_ [100]. With the strong gradient perpendicular to the leg, the nonlinear gradient coil is a promising way to deliver short‐diffusion‐time microstructure imaging of the leg muscle [101, 102].

## 6 Conclusions

This work presents an analysis of the inside‐out nonlinear gradient coil for DWI, develops corresponding prostate DWI protocols, and reveals improvements in image quality brought about by locally strong gradient for prostate DWI. This provides an alternative for high‐performance DWI targeted for specific organs and paves the way for accessible strong gradients of the prostate.

## Funding

This work was supported by National Institutes of Health, CA264851.

## Conflicts of Interest

The authors declare no conflicts of interest.

## Supporting information


**Table S1:** Information of patients and lesions. Lesion size is the length in the largest dimension.
**Figure S1:** Background *B*_0_ map with the nonlinear gradient in the scanner bore. A sagittal slice on the left of the coil is shown. GRE is the reference image for the bottle phantom (a) and the leg (b), and the EPI distortion is in accordance with the measured Δ*B*_0_ map, which shows considerable distortion on the superior–inferior edge and negligible distortion in the middle. Brightened EPI highlights ghosts, and the ghost‐to‐signal ratio is not considerable. (c) As in‐plane acceleration (GRAPPA) factor increases, distortion in the phase encoding direction is mitigated, along with less signal pile‐up. Ry = 3 is used in this study as higher acceleration shows similar distortion but has lower SNR.
**Figure S2:** Repeated GRE with a regular interval at each phase encoding step was used for characterization of eddy currents after the nonlinear gradient waveform.
**Figure S3:** Eddy current maps on a coronal slice, which show the through‐slice eddy currents for an axial slice of prostate imaging. (a) Selected time frames of the dynamic eddy currents show Larmor frequency offset changing with time. For a typical axial slice, the intra‐slice difference of Larmor frequency is only around 1 Hz, suggesting negligible through‐slice dephasing. In addition, as the slow decay of this component implies it arises from coupling with the superconducting magnet, the slight spatial variation may be artifactual. The changing Larmor frequency results in small changes in slice selection over the acquisition, which leads to imperfect cancelation of the previously acquired background field. Consistent with that interpretation, spatial variation in the slow decaying component was greater with weaker slice selection gradient strength. Further characterization was not pursued due to the negligible amplitude of this variation, on the order of single μT/m. (b) Principal component analysis reveals the dominating component as Larmor frequency offset and other minor higher‐order components.
**Figure S4:** The effect of denoising and deranging on unaveraged DWI.
**Figure S5:** An example of nonlinear‐gradient DWI at *b* = 3000 s/mm^2^ shown as original and normalized, which are similar in the prostate region. The normalized DWI is an extrapolated result with a uniform *b*‐value using an ADC model: $S_{\text{normalized}}\left(b_{\text{uniform}}\right)=S_{\text{original}}\left(b=0\right)\cdot {\left[S_{\text{original}}\left(b_{\text{nonuniform}}\right)/S_{\text{original}}\left(b=0\right)\right]}^{b_{\text{uniform}}/b_{\text{nonuniform}}}$.
**Figure S6:** Nonlinear gradient maps derived from the total field ($B_{\text{total}}=\sqrt{B_{r}^{2}+{\left(B_{z}+3\right)}^{2}}-3$) versus the longitudinal field ($B_{z}$) show differences only at certain parts of the coil edges due to the concomitant field ($B_{r}$), visible on the scale of 1 mT/m. No visible difference is observed at the prostate location, demonstrating that concomitant fields have minimal impact on prostate DWI, and that total field and longitudinal field mapping yield similar nonlinear gradient maps, especially negligible in the prostate region (< 0.01 mT/m).

## Data Availability

The data that support the findings of this study are available from the corresponding author upon reasonable request.
