# Supplementary material for: Strong‐Gradient Diffusion‐Weighted Imaging of Prostate Cancer Using an Inside‐Out Nonlinear Gradient Coil
Source: Magn Reson Med. 2026 Jan 8;95(5):2671–88. doi: 10.1002/mrm.70252 (PMC12962214; doi:10.1002/mrm.70252)
Supplement: Supplementary file 1 — Table S1: Information of patients and lesions. Lesion size is the length in the largest dimension. Figure S1: Background B 0 map with the nonlinear gradient in the scanner bore. A sagittal slice on the left of the coil is shown. GRE is the reference image for the bottle phantom (a) and the leg (b), and the EPI distortion is in accordance with the measured ΔB 0 map, which shows considerable distortion on the superior–inferior edge and negligible distortion in the middle. Brightened EPI highlights ghosts, and the ghost‐to‐signal ratio is not considerable. (c) As in‐plane acceleration (GRAPPA) factor increases, distortion in the phase encoding direction is mitigated, along with less signal pile‐up. Ry = 3 is used in this study as higher acceleration shows similar distortion but has lower SNR. Figure S2: Repeated GRE with a regular interval at each phase encoding step was used for characterization of eddy currents after the nonlinear gradient waveform. Figure S3: Eddy current maps on a coronal slice, which show the through‐slice eddy currents for an axial slice of prostate imaging. (a) Selected time frames of the dynamic eddy currents show Larmor frequency offset changing with time. For a typical axial slice, the intra‐slice difference of Larmor frequency is only around 1 Hz, suggesting negligible through‐slice dephasing. In addition, as the slow decay of this component implies it arises from coupling with the superconducting magnet, the slight spatial variation may be artifactual. The changing Larmor frequency results in small changes in slice selection over the acquisition, which leads to imperfect cancelation of the previously acquired background field. Consistent with that interpretation, spatial variation in the slow decaying component was greater with weaker slice selection gradient strength. Further characterization was not pursued due to the negligible amplitude of this variation, on the order of single μT/m. (b) Principal component analysis reveals the dominati [file MRM-95-2671-s001.pdf]

ID	Age	Prostate Size	PI-RADS	Lesion Location	Lesion Size
**1**	69	4.9 × 5.0 × 3.6 cm^3	4	right posterior medial mid gland	0.7 cm
			3	right base anteromedial	1.2 cm
**2**	64	4.8 × 3.9 × 4.8 cm^3	4	left posterior mid lateral	1.3 cm
**3**	61	4.2 × 5.3 × 4.0 cm^3	3	left peripheral zone at the base	1.3 cm
**4**	82	4.0 × 4.4 × 3.7 cm^3	5	left posterior peripheral zone	2.0 cm
**5**	76	6.3 × 3.9 × 5.8 cm^3	5	right prostatic base transition zone	3.0 cm
**6**	60	4.1 × 4.1 × 3.0 cm^3	4	right posterior peripheral zone	0.8 cm
**7**	65	5.5 × 4.4 × 3.4 cm^3	5	right transition zone	1.7 cm
**8**	87	4.6 × 4.8 × 4.9 cm^3	5	right peripheral zone	2.9 cm
			5	throughout the transition zone	3.8 cm

Table S(1). Information of patients and lesions. Lesion size is the length in the largest dimension.

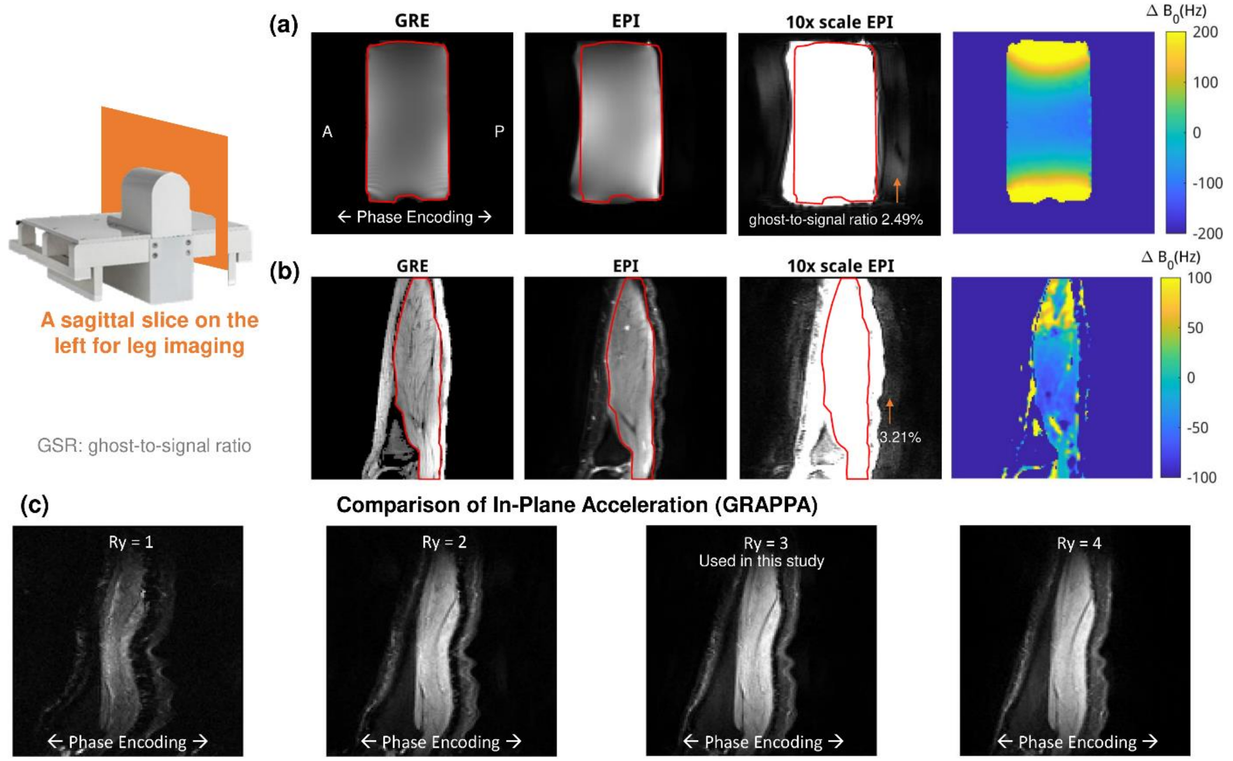

Figure S(1). Background B_0 map with the nonlinear gradient in the scanner bore. A sagittal slice on the left of the coil is shown. GRE is the reference image for the bottle phantom (a) and the leg (b), and the EPI distortion is in accordance with the measured ΔB_0 map, which shows considerable distortion on the superior-inferior edge and negligible distortion in the middle. Brightened EPI highlights ghosts, and the

ghost-to-signal ratio is not considerable. (c) As in-plane acceleration (GRAPPA) factor increases, distortion in the phase encoding direction is mitigated, along with less signal pile-up. Ry = 3 is used in this study as higher acceleration shows similar distortion but has lower SNR.

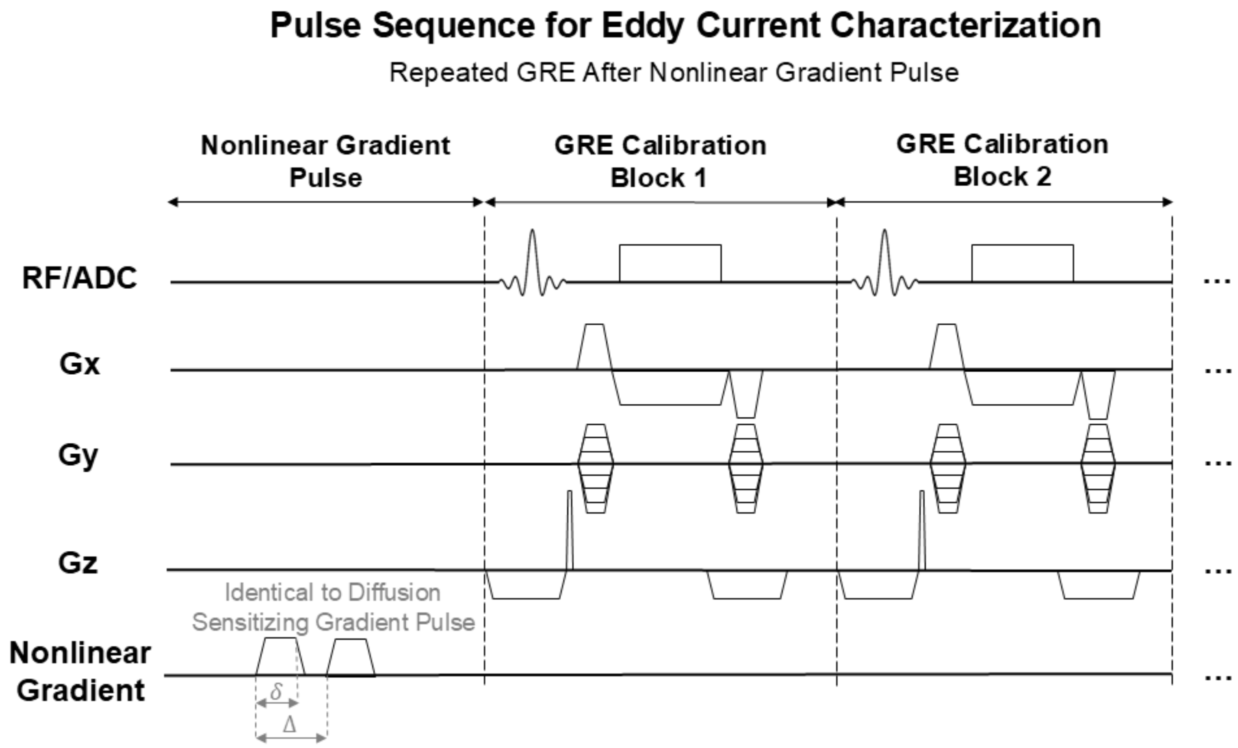

Figure S(2). Repeated GRE with a regular interval at each phase encoding step was used for characterization of eddy currents after the nonlinear gradient waveform.

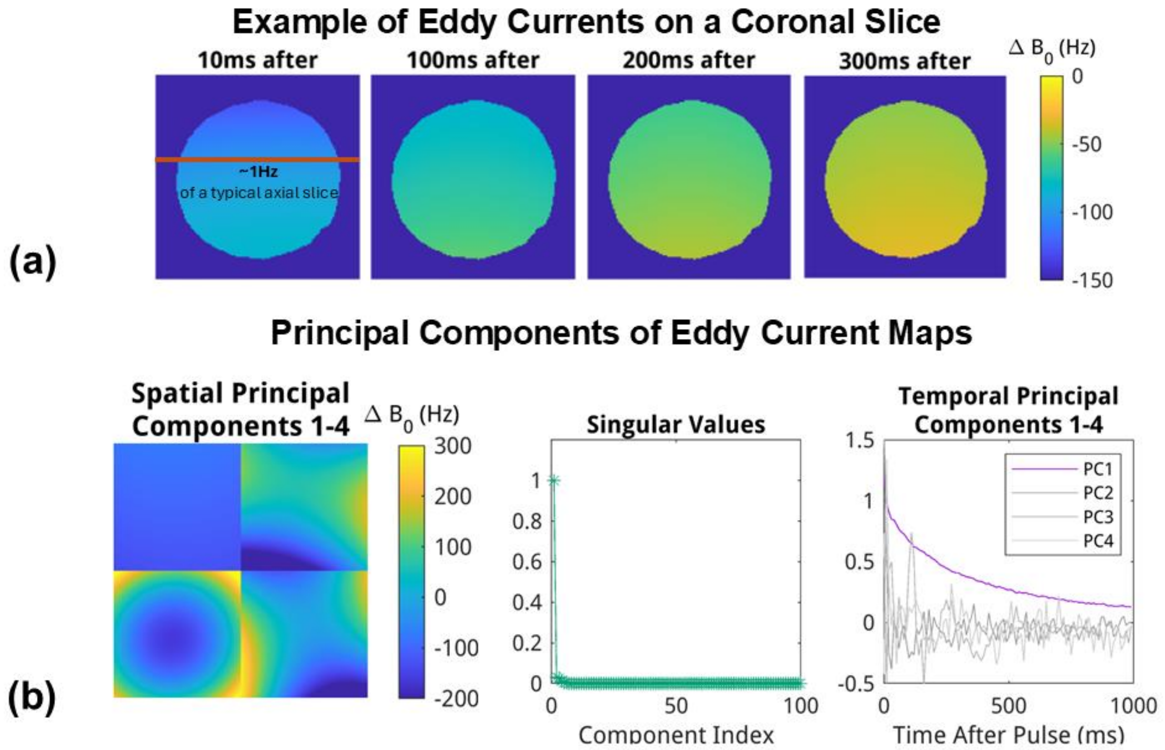

Figure S(3). Eddy current maps on a coronal slice, which show the through-slice eddy currents for an axial slice of prostate imaging. (a) Selected time frames of the dynamic eddy currents show Larmor frequency offset changing with time. For a typical axial slice, the intra-slice difference of Larmor frequency is only around 1 Hz, suggesting negligible through-slice dephasing. In addition, as the slow decay of this component implies it arises from coupling with the superconducting magnet, the slight spatial variation may be artifactual. The changing Larmor frequency results in small changes in slice selection over the acquisition, which leads to imperfect cancellation of the previously acquired background field. Consistent with that interpretation, spatial variation in the slow decaying component was greater with weaker slice selection gradient strength. Further characterization was not pursued due to the negligible amplitude of this variation, on the order of single μT/m. (b) Principal component analysis reveals the dominating component as Larmor frequency offset and other minor higher-order components.

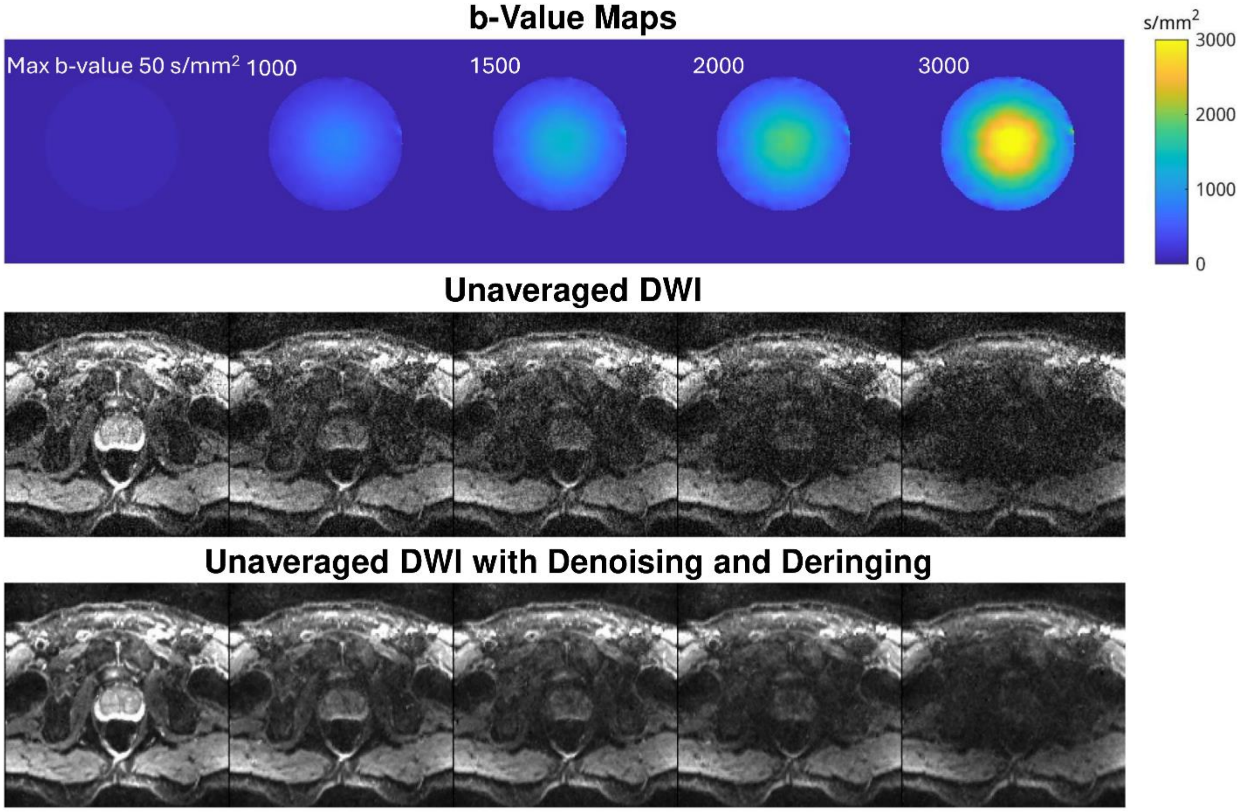

Figure S(4). The effect of denoising and deringing on unaveraged DWI.

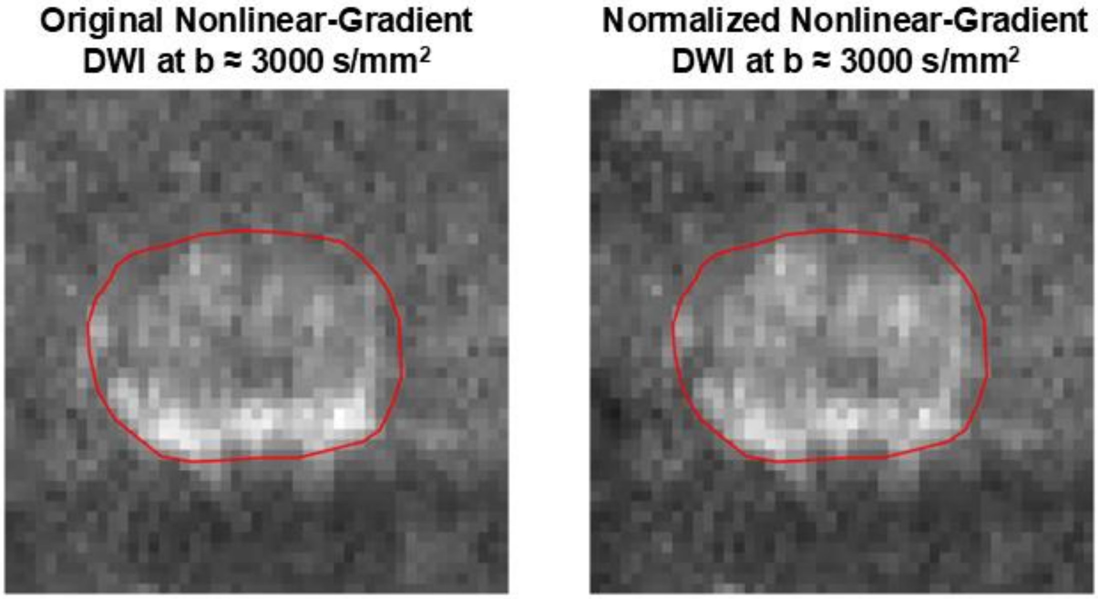

Figure S(5). An example of nonlinear-gradient DWI at b = 3000 s/mm^2 shown as original and normalized, which are similar in the prostate region. The normalized DWI is an extrapolated result with a uniform b-

value using an ADC model: $S_{normalized}(b_{uniform}) = S_{original}(b = 0) \cdot [S_{original}(b_{nonuniform})/S_{original}(b = 0)]^{b_{uniform}/b_{nonuniform}}$

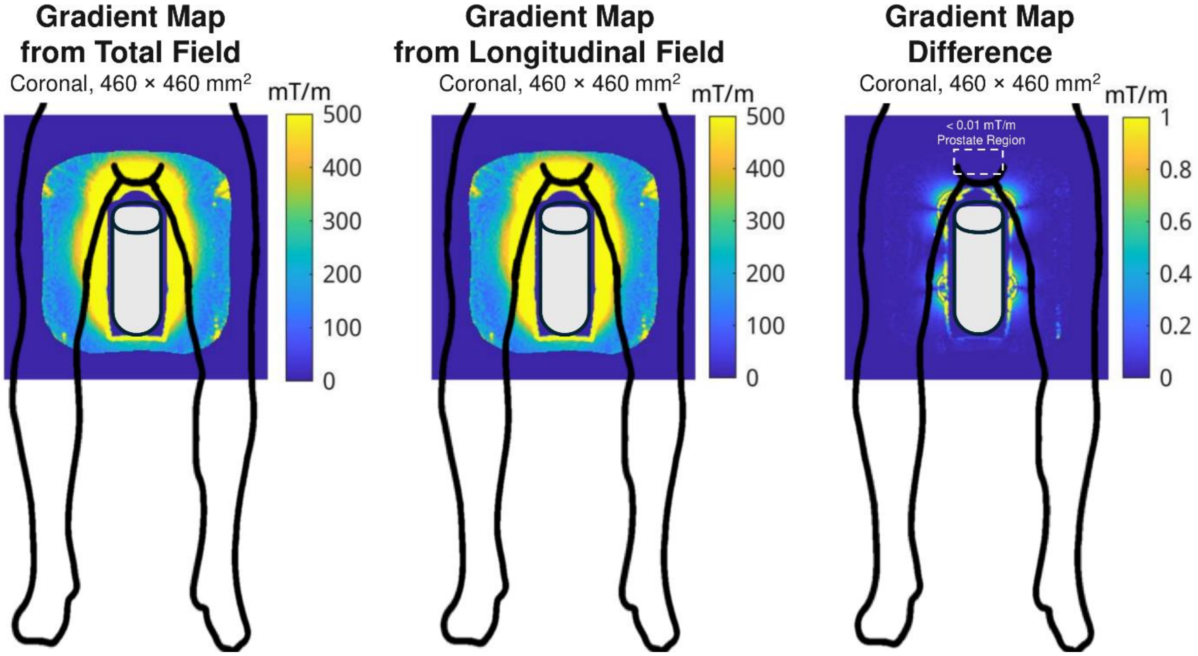

Figure S(6). Nonlinear gradient maps derived from the total field ($\Delta B_{total} = \sqrt{B_r^2 + (B_z + 3)^2} - 3$) versus the longitudinal field (B_z) show differences only at certain parts of the coil edges due to the concomitant field (B_r), visible on the scale of 1 mT/m. No visible difference is observed at the prostate location, demonstrating that concomitant fields have minimal impact on prostate DWI, and that total field and longitudinal field mapping yield similar nonlinear gradient maps, especially negligible in the prostate region (< 0.01 mT/m).
